# Supplementary material for: Mutual inclusivity improves decision-making by smoothing out choice’s competitive edge
Source: bioRxiv. 2023 Jul 24:2023.05.12.540529. Preprint. [Version 5] doi: 10.1101/2023.05.12.540529 (PMC10327072; doi:10.1101/2023.05.12.540529)
Supplement: Supplementary file 1 [file media-1.pdf]

Supplementary Materials

In the Supplementary Materials, we included (a) results of additional mixed-effect models of choice conflict in Study 1 and 2 with first-choice RT and accuracy included as predictors (**Table S1-S2**); (b) supplementary plots for Study 3A-B (**Figure S1-S4**); and (c) supplementary plots for Study 4 (**Figure S5-S6**).

Table S1. Mixed-effect model of conflict in Study 1 when including first-choice RT and accuracy

	**Conflict**		
Predictors	***Estimates***	***CI***	***p***
Intercept	2.36	2.23 – 2.50	**<0.001**
RT	0.09	0.05 – 0.13	**<0.001**
Accuracy	-0.09	-0.13 – -0.05	**<0.001**
Inclusivity	-0.44	-0.59 – -0.28	**<0.001**
OV	19.55	10.17 – 28.93	**<0.001**
OV^2	11.11	6.56 – 15.66	**<0.001**
RV	-0.03	-0.06 – 0.00	0.075
Trial Num	0.01	-0.03 – 0.05	0.696
Incl by OV	-17.43	-25.30 – -9.56	**<0.001**
Incl by OV^2	-5.03	-10.05 – -0.02	**0.049**
Excl by RV	-0.01	-0.06 – 0.05	0.836

Table S2. Mixed-effect model of conflict in Study 2 when including first-choice RT and accuracy

	**Conflict**		
Predictors	***Estimates***	***CI***	***p***
Intercept	2.17	2.08 – 2.27	**<0.001**
RT	0.13	0.09 – 0.16	**<0.001**
Accuracy	-0.00	-0.04 – 0.03	0.837
Inclusivity	-0.08	-0.15 – -0.01	**0.033**
OV	39.80	31.54 – 48.07	**<0.001**
OV^2	18.96	14.98 – 22.93	**<0.001**
RV	-0.05	-0.07 – -0.02	**0.001**
Trial Num	0.01	-0.03 – 0.04	0.695
Incl by OV	4.40	-1.54 – 10.34	0.147
Incl by OV^2	-3.34	-7.97 – 1.29	0.158
Excl by RV	0.03	-0.01 – 0.07	0.170

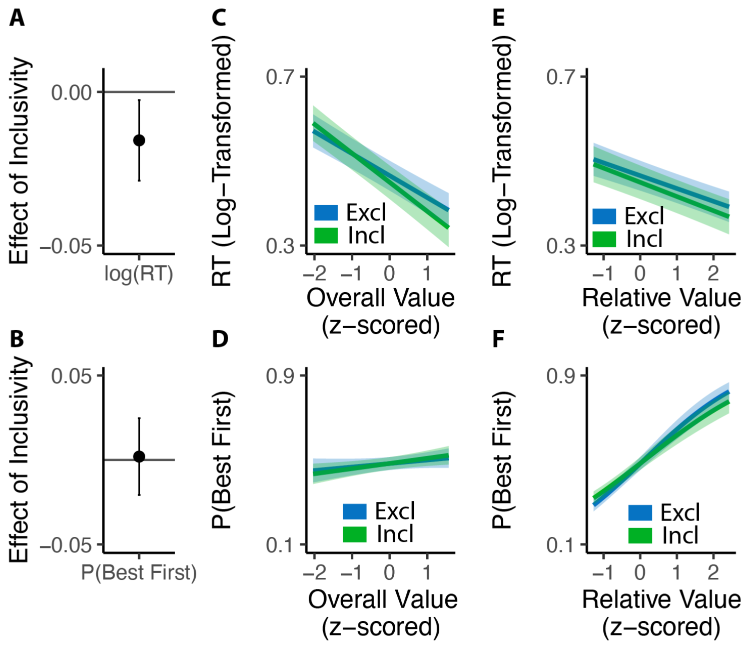

Figure S1. Influence of inclusivity on choice behavior in Study 3A. (A-F) The effect of inclusivity on choice behavior is consistent with the behavioral patterns in Study 1 (**Figure 2**).

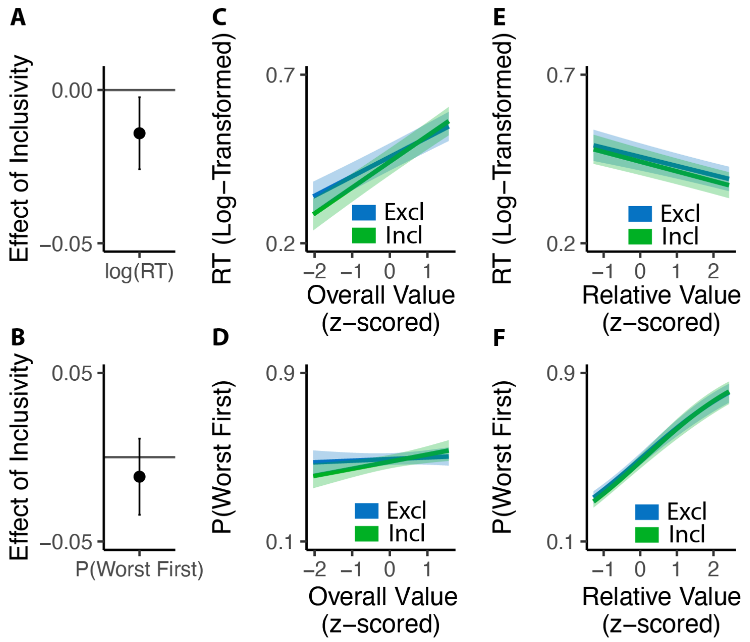

Figure S2. Influence of inclusivity on choice behavior in Study 3B. (A-F) The effect of inclusivity on choice behavior is consistent with the behavioral patterns in Study 2 (**Figure 5**).

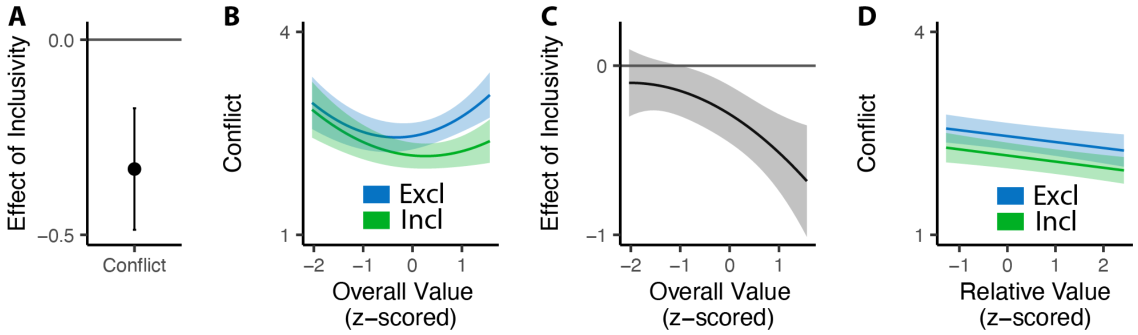

Figure S3. Influence of inclusivity on choice conflict in Study 3A. (A-D) The effect of inclusivity on choice conflict replicates the results in Study 1 (**Figure 4**).

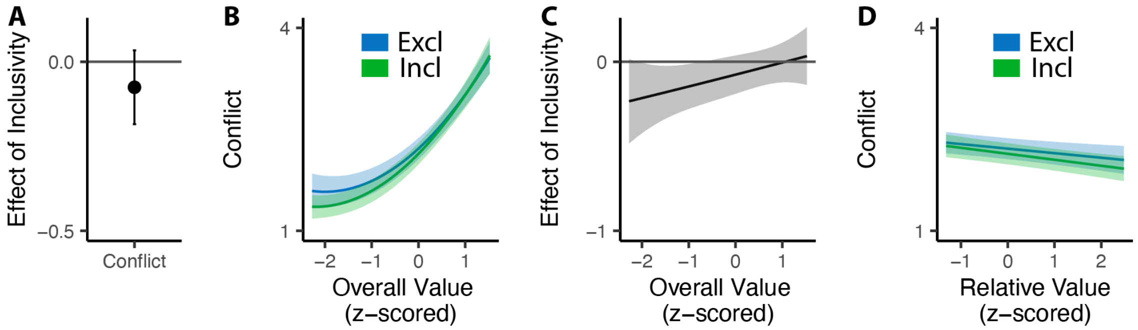

Figure S4. Influence of inclusivity on choice conflict in Study 3B. (A-D) The effect of inclusivity on choice conflict replicates the results in Study 2 (**Figure 6**).

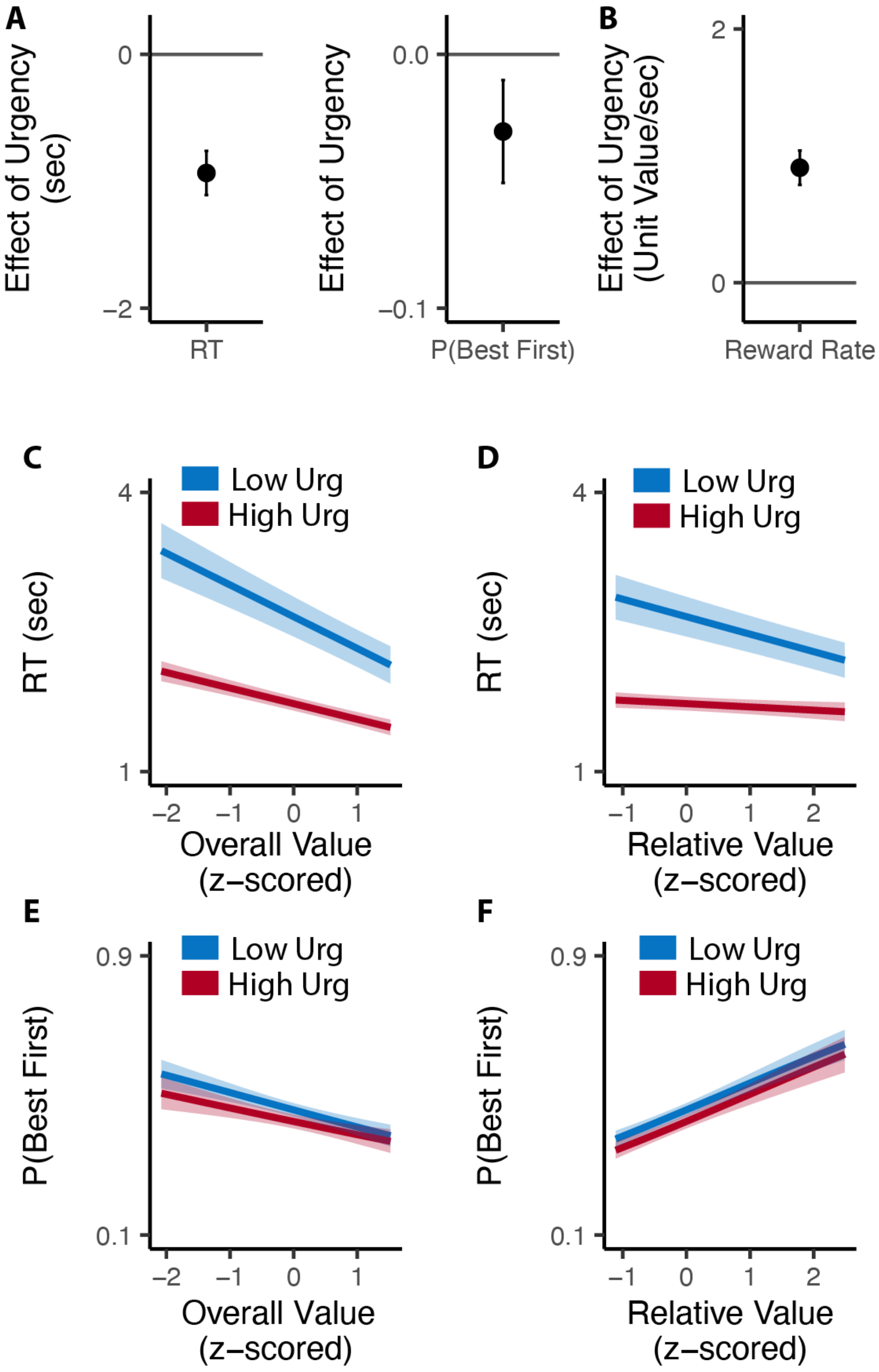

Figure S5. Influence of urgency on choice behavior in Study 4. (A-B) People made faster and less accurate choices with high choice urgency, leading to higher reward rate. **(C-D)** Choice urgency similarly modulates the influence of overall value and relative value on choice reaction time. **(E-F)** The effects of overall value and relative value on choice accuracy do not differ across high and low choice urgency.

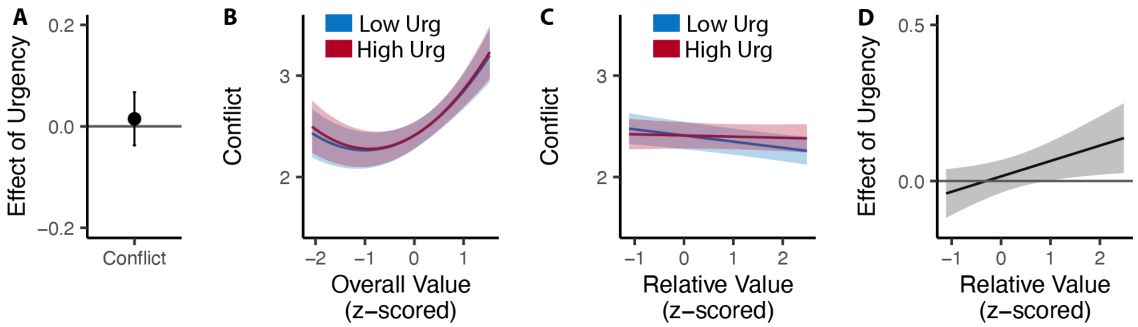

Figure S6. Influence of urgency on choice conflict in Study 4. (A) High choice urgency does not influence overall choice conflict. **(B)** Choice conflict increases when the overall value is extremely high or low, regardless of choice urgency. **(C)** With high choice urgency, higher relative value does not reduce choice conflict. **(D)** Higher choice urgency increases choice conflict when the relative value is larger.
